# A retrospective cohort study on cassava food poisoning, Santa Cruz, Davao del Sur, Philippines, October 2015

**DOI:** 10.5365/wpsar.2017.8.1.010

**Published:** 2018-10-25

**Authors:** Johnette Peñas, Vikki Carr de los Reyes, Ma. Nemia Sucaldito, Denisse Lou Manalili, Herdie Hizon, Rio Magpantay

**Affiliations:** a Department of Health, Philippines.

## Abstract

**Objective:**

On 2 October 2015, the Event-Based Surveillance and Response Unit of the Department of Health (DOH), Philippines received a report of foodborne illness cases in Santa Cruz, Davao del Sur. A team from DOH was sent to conduct an investigation to identify the implicated source and determine risk factors.

**Methods:**

A retrospective cohort study was done. A suspect case was defined as a previously well individual in Compound A, Santa Cruz who developed abdominal pain, headache, dizziness, diarrhoea or vomiting on either 1 or 2 October 2015. A confirmed case was a suspect case positive for cyanide in urine. Family members who prepared the food were interviewed. Urine specimens were collected to test for thiocyanate, and cassava tuber and soil samples were tested for cyanide and other chemicals.

**Result:**

Fourteen cases with two deaths were identified (case fatality ratio: 14%). All cases consumed cassava on 1 October 2015 except for one child who spat it out. Urine samples were all negative (36, 100%) for thiocyanate so there were no confirmed cases. The cassava sample had a cyanide level of 68.94 ug/g and was identified as bitter cassava, also known as a potentially dangerous kind. Insufficient food preparation was noted. In the retrospective cohort study, intake of cassava (RR = 208, 95% CI: 19.94–2169.32) was associated with the illness.

**Discussion:**

This study identified insufficiently processed cassava root crop as the source of the foodborne illness. The cassava consumed was the bitter variety that contains greater than 50 ug/g of hydrogen cyanide and requires thorough preparation before consumption. Community education was provided on identifying and preparing cassava appropriately.

## Introduction

Cassava is the third most important source of calories in the tropics. (*1*) Millions of people depend on cassava in Africa, Latin America and Asia both for food security and income generation. (*2*) In the Philippines, cassava is advocated as an alternative staple to rice under the Department of Agriculture – Food Staple Self-Sufficiency Program. (*3*)

However, several cases of acute poisoning, some leading to death, following consumption of a cassava-based meal have been reported. (*4*–*7*) Common symptoms include dizziness, nausea, headache, abdominal pain and diarrhoea. (*8*) This is due to the toxic chemical linamarin which occurs in varying amounts in all parts of the cassava plant. Ingested linamarin can release cyanide in the gut during digestion, causing illness and sometimes death. (*9*)

Cassava is generally classified into two main types: sweet cassava and bitter cassava. Cassava roots with less than 50 ug/g hydrogen cyanide on fresh weight basis are considered sweet; above this level, cassava roots are considered bitter. (*10*) Sweet cassava roots can be made safe to eat by peeling and thoroughly cooking. For bitter cassava, one traditional way to effectively reduce its cyanide content is by peeling the root crop followed by grating, prolonged soaking (18–24 hours), squeezing and thorough cooking. (*11*)

On 2 October 2015, the Davao Department of Health (DOH) Regional Office, reported to the Event-Based Surveillance and Response Unit of DOH several foodborne illness cases in Santa Cruz, Davao del Sur. Santa Cruz is a municipality located in Davao Region, which is part of the Mindanao group of islands. The municipality is situated about 988 km south-eastern of the Philippine capital Manila.

A team from DOH was sent to conduct an investigation to identify the implicated source and determine risk factors.

## Methods

### Epidemiological investigations

A suspect case was defined as a previously well individual in Compound A, a residential area shared by seven families in Santa Cruz, who developed abdominal pain, headache, dizziness, diarrhoea or vomiting on either 1 or 2 October 2015. A confirmed case was defined as a suspect case positive for cyanide in the urine. Case finding was done by reviewing medical records in Cereville Medical Clinic, Davao del Sur Provincial Hospital and Southern Philippines Medical Center.

A retrospective cohort study was done in Compound A. All residents were interviewed using a standard questionnaire comprising questions on demographics, symptoms, hygiene practices and 24-hour food recall. A parent was interviewed for the two fatal cases, and children were interviewed along with their parents for all other cases. We calculated relative risks (RR), 95% confidence intervals (CI) and *p* values using Epi Info 3.5.4. Risk factors approaching significance (*P* < 0.2) in bivariate analysis were retained for multivariable logistic regression using a forward stepwise procedure.

### Laboratory examination

Twenty-eight blood specimens (from nine ill and 19 not ill people) were collected to measure sulfhaemoglobin and methaemoglobin levels to determine exposure to oxidizing drugs or toxins. Thirty-six urine samples (from 10 ill and 26 not ill people) were collected for thiocyanate testing to identify the presence of cassava derivatives. (*12*)

Cassava tuber from the same cassava plant consumed by the families and soil samples from where it was planted were collected to test for cyanide and pesticides. A cyanide level of more than 50 ug/g would classify the cassava as bitter type. (*10*) All samples were collected on 6 October 2015. Blood specimens were sent to East Avenue Medical Center, Quezon City. Urine, cassava and soil samples were analysed at Chempro Analytical Services Laboratories Inc., Pasig City.

### Environmental investigation

A site visit was conducted in Compound A to identify the circumstances surrounding the event. We interviewed family members who cooked the cassava crop about its source and preparation. We also inspected the source-farm where the raw cassava was harvested. Information on the variety of cassava was elicited from the municipal agriculturist. We asked the farmer about pesticides and other chemicals used in growing cassava.

## Results

### Cases

**Fig. 1:**
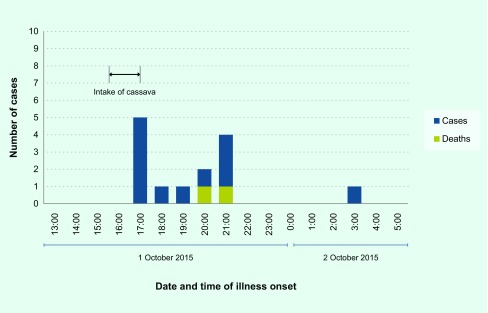
Number of cases by date and time of illness onset, cassava poisoning, Compound A, Santa Cruz, Davao del Sur, October 2015 (*n* = 14)

Fourteen cases were identified. The incubation period ranged from one hour to 12 hours (median = 3.25 hours). The earliest onset of illness was at 17:00, one hour after intake of the cassava; this was the peak of the epidemic curve (**Fig. 1**). The last case was a 1-year-old child who was fed by her mother with two spoonfuls of cassava, which the child spat out. Most of the cases had abdominal pain (13/14, 93%) followed by diarrhoea (4/14, 29%), headache (3/14, 21%), dizziness (3/14, 21%) and vomiting (3/14, 21%). Thirteen cases sought medical care, but three refused hospital admission. Two cases were referred for further management but died before they were transported to another facility (case fatality ratio = 14%).

Cases ranged in age from 1 to 28 years (median = 11 years)*;* seven (50%) were males. The most affected age group was 0–4 years old. All cases were from two families in Compound A. All of them ate cassava before the onset of illness except for one child who spat it out.

### Profile of deaths

Ages of the two fatalities were 4 and 2 years. Both were males from the same household. The older child died three hours after manifesting symptoms. The other child was pronounced dead five hours after onset of illness. Both had consumed four slices of boiled cassava.

### Retrospective cohort study

All (65, 100%) residents of Compound A were interviewed. Seventeen (25%) ate boiled cassava. On bivariate analysis, we found that age 0–5 years (RR = 4.91, 95% CI: 2.16–11.18) and intake of cassava (RR = 39.81, 95% CI: 5.64–280.99) were associated with the illness. Handwashing before eating (RR = 0.24, 95% CI: 0.11–0.52) was found to be inversely associated with the event. After multivariable analysis, intake of cassava (RR = 208, 95% CI: 19.94–2169.32) was the only risk factor associated with the illness (**Table 1**).

**Table 1:** Factors associated with cassava poisoning, Compound A, Santa Cruz, Davao del Sur, October 2015

Variables	Sick (*n* = 14) *n*(%)	Not sick (*n* = 51) *n*(%)	*P*-value	Crude RR (95% CI)	Adjusted RR** (95% CI)
Male	7 (50%)	16 (31%)	0.20	1.83 (0.73–4.56)	1.28 (0.31–5.21)
1–5 years old	7 (50%)	4 (9%)	< 0.01	4.91(2.16–11.18)	4 964 152.23 (0.00 – > 1.0E12)
Food eaten*					
Fish	14 (100%)	49 (96%)	0.61	Undefined	-
Bread	2 (14%)	6 (12%)	0.55	1.19 (0.32–4.36)	-
Egg	1 (7%)	4 (8%)	0.71	0.92 (0.15–5.69)	-
Noodles	1 (7%)	0 (0%)	0.21	4.92 (3.03–8.00)	-
Monggo	0 (0%)	2 (4%)	0.61	0 (undefined)	-
Cassava	13 (93%)	3 (6%)	< 0.01	39.81 (5.64–280.99)	208 (19.94–2169.32)
History of cassava intake	11 (79%)	46 (90%)	0.23	0.51 (0.18–1.46)	-
Hygiene					
Washed hands before eating	11 (79%)	50 (98%)	0.03	0.24 (0.11–0.52)	0.14 (0.00–11.50)
Used soap in hand washing	14 (100%)	50 (98%)	-	-	-
Used spoon and fork	0 (0%)	5 (10%)	0.28	0.00 (undefined)	-
Ate food while hot	13 (93%)	42 (82%)	0.31	2.36 (0.35–16.11)	-
Washed hands after toilet use	13 (93%)	51 (100%)	0.22	0.20 (0.13–0.33)	-

CI, confidence interval; RR, relative risk

* May have more than one response

** Risk factors approaching significance (*P* < 0.2) in bivariate analysis were included in multivariable logistic regression using a forward stepwise procedure.

### Environmental investigation

The cassava was harvested by the father in Family A and was shared with Family B. The cassava was prepared by the father in Family A and by a daughter in Family B. The cassava was peeled, washed and boiled in water for one hour. No other ingredients were added. Family A shared the cooked cassava among themselves. Family B shared it with Families C and D.

The farmer who planted the cassava claimed that no fertilizer was used. Sweet and bitter varieties were grown in the field, and all were intended to be processed into animal feed and not for household consumption. The cassava had been harvested without permission.

### Laboratory examination

Sulfhaemoglobin was not detected in 28 clinical specimens. Methaemoglobin was not detected in eight (29%) individuals and the rest were below 0.5 g/dL (normal limit). Urine tests for thiocyanate were all negative (36, 100%).

The cassava sample had a cyanide level of 68.94 ug/g. Organochloride and organophosphate pesticides were not detected in cassava and soil samples.

## Discussion

This foodborne outbreak was most likely due to consumption of insufficiently processed bitter cassava. The cassava sample had a cyanide level of 68.94 ug/g, which classified it as the bitter variety. Although there were no confirmed cases, all except one case had a history of boiled cassava intake, and eating cassava was a significant risk factor (RR = 208, 95% CI: 19.94–2169.32). Signs and symptoms manifested by cases were consistent with those reported in other studies. (*4*, *5*, *8*) Insufficient processing of cassava was attributed to this outbreak as it has been in other outbreaks. (*6*, *7*)

Cassava varieties are usually differentiated from one another by their morphological characteristics such as colour of stems, petioles, leaves and tubers. Generally, the bitter varieties of cassava are recognized by dark leaves and stems, often tending to be reddish in colour, whereas the sweet varieties have light-green leaves and stems. (*13*) This does not apply in the Philippines where petioles and stems of several varieties of sweet cassava are pink or red. The two plants are extremely difficult to distinguish in the field, and distinction between them rests upon the content of hydrocyanic acid. (*14*) This could explain why the father harvested the bitter variety of cassava instead of the sweet type. The cassava he harvested was meant as animal feed.

The cases’ sulfhaemoglobin and methaemoglobin levels were insignificant; organochloride and organophosphate pesticides were not detected in cassava or soil samples. This rejects a possible relationship of other chemicals to these food poisonings. However, it is also likely that sulfhaemoglobin and methaemoglobin tested normal or within limit because no samples were collected from the two fatalities who might have had higher exposures.

This study has some limitations. There was no leftover boiled cassava for testing, and no specimens were collected from the two fatalities. All urine samples were negative for cyanide, and there were no laboratory-confirmed cases. However, as the urine samples were collected four days after the incident, it is possible that thiocyanate in the urine was not detected because most of cyanide by-products leave the body within 24 hours after exposure. (*15*) Despite these limitations, valid statistical and cause-and-effect association strongly suggest cassava as the cause of this foodborne outbreak.

As a result of this outbreak, a DOH memorandum on health advice on common plants containing toxins was disseminated and reiterated by the Southern Philippines Medical Center and Davao DOH Regional Office. Health advice includes information on early signs of acute cyanide poisoning, management and the recommended processing (peeling outer skin, grating, soaking in water and squeezing) and cooking regardless of the variety of cassava. Community education was conducted in villages of Santa Cruz municipality. Public awareness on cassava varieties and its proper preparation is essential to prevent this kind of incident.
